# Welding Defect Monitoring Based on Multi-Scale Feature Fusion of Molten Pool Videos

**DOI:** 10.3390/s24206561

**Published:** 2024-10-11

**Authors:** Chenbo Shi, Lei Wang, Changsheng Zhu, Tengyue Han, Xiangyu Zhang, Delin Wang, Chun Zhang

**Affiliations:** 1 College of lntelligent Equipment, Shandong University of Science and Technology, Taian 271019, China; skd996523@sdust.edu.cn (C.S.); 202283230040@sdust.edu.cn (L.W.); zcs@sdust.edu.cn (C.Z.); 202383230063@sdust.edu.cn (X.Z.); 202383230017@sdust.edu.cn (D.W.); 2 Beijing Botsing Technology Co., Ltd., Beijing 100176, China; hty@botsing.com

**Keywords:** molten pool, welding defect monitoring, dynamic characteristics, multi-scale feature fusion, light spots

## Abstract

Real-time quality monitoring through molten pool images is a critical focus in researching high-quality, intelligent automated welding. However, challenges such as the dynamic nature of the molten pool, changes in camera perspective, and variations in pool shape make defect detection using single-frame images difficult. We propose a multi-scale fusion method for defect monitoring based on molten pool videos to address these issues. This method analyzes the temporal changes in light spots on the molten pool surface, transferring features between frames to capture dynamic behavior. Our approach employs multi-scale feature fusion using row and column convolutions along with a gated fusion module to accommodate variations in pool size and position, enabling the detection of light spot changes of different sizes and directions from coarse to fine. Additionally, incorporating mixed attention with row and column features enables the model to capture the characteristics of the molten pool more efficiently. Our method achieves an accuracy of 97.416% on a molten pool video dataset, with a processing time of 16 ms per sample. Experimental results on the UCF101-24 and JHMDB datasets also demonstrate the method’s generalization capability.

## 1. Introduction

GMAW (Gas Metal Arc Welding) is widely used in various modern manufacturing industries, such as shipbuilding and storage tank construction, due to its advantages in automation and mechanization [1]. Despite the significant advancements in welding technology, the advanced manufacturing industry continues to demand higher welding production efficiency, intelligent automation, and superior welding quality. However, various welding defects remain unavoidable in actual welding processes. Among these, porosity is a critical issue affecting the quality of welded structures. The presence of porosity reduces the cross-sectional area at the welded joints, leads to uneven stress distribution, and severely impacts the quality of the weld. Therefore, monitoring porosity defects during welding is an urgent problem that needs to be addressed.

Welding is a dynamic, interactive, and nonlinear process. Experienced welders can improve weld quality and reduce defects by observing the molten pool and making real-time adjustments during the welding process [2]. However, extended observation of the molten pool can lead to welder fatigue, making it difficult to detect defects promptly and adjust the process accordingly. Additionally, welding produces irritating gases that pose health risks to welders. As a result, automated monitoring of the welding process using molten pool images to detect defects has become a research focus among scholars worldwide, aiming to replace the manual observation of the molten pool with automated methods.

When using features from molten pool images to identify welding defects, the critical challenge is linking the image features to the welding defects and establishing a mapping model from molten pool image features to welding defects. Research based on molten pool images can be broadly categorized into two types: one focusing on welding defect detection using single-frame molten pool images, and the other utilizing sequences of molten pool images (i.e., molten pool videos). For single-frame molten pool image analysis, one approach involves performing a multi-level statistical analysis of the geometric features of the molten pool, such as area, shape, and aspect ratio, to determine the state of the molten pool when defects occur [3,4,5,6,7,8]. This method, based on geometric feature information, is highly interpretable. However, this approach requires extensive statistical analysis, making it time-consuming. Moreover, due to variations in welding techniques and types, molten pool images may differ significantly, making it challenging to extend these geometric feature-based methods to other images. With the continuous advancement of deep learning technology, data-driven approaches like deep learning have been widely applied in image classification, object detection, speech recognition, and natural language processing [9,10,11,12]. In the context of molten pool image analysis, deep learning allows for the direct, end-to-end adaptive learning and extraction of molten pool features, replacing manual feature extraction. This approach not only enhances efficiency but also achieves excellent results in defect recognition [13,14]. To address the issue of CNNs (Convolutional Neural Networks) often being perceived as black boxes and the lack of large datasets of welding defects, Di Wu et al. [15] proposed a method that combines deep learning-extracted features with manually designed geometric features for prediction. This approach improves model accuracy and enhances the interpretability of the network. In studies involving molten pool image sequences, some works [1,16] utilize LSTM (Long Short-Term Memory) networks [17] to capture the differential features of the molten pool before and after changes during the welding process. These studies infer future frames of molten pool images and identify welding defects in those future frames, achieving early quality warnings.

In [2], Tianyuan Liu et al. proposed a CNN-LSTM model for online defect recognition in CO_2_ welding. This model stretches the extracted features into two-dimensional representations, treating each row as a time series. By leveraging the strengths of LSTM in processing sequential data, the model effectively selects features in the spatial dimension, enabling accurate welding defect recognition. Although this approach utilizes LSTM, it only processes single-frame molten pool images and does not take advantage of the dynamic information inherent in the welding process. In contrast, Jun Lu et al. [1] developed a MPOM (Molten Pool Online Monitoring) model for monitoring the welding process, incorporating prediction and classification networks. The prediction model uses LSTM to capture the differences in molten pool states caused by temperature variations, allowing for predicting future molten pool shapes up to 10 time intervals in advance. These predicted future frames are then used for welding defect classification, highlighting the importance of molten pool features during the welding process for defect identification. However, research focusing on using dynamic features from the welding process for defect detection remains scarce.

Traditional RNNs (Recurrent Neural Networks) were introduced to handle sequential tasks, but they still need to be improved regarding gradient explosion and long-term dependency issues [18]. LSTM [17] addresses the gradient vanishing and explosion problems often occurring in long-sequence processing. However, LSTM models have a large number of parameters and face challenges when dealing with even more extended sequences. The C3D (3D Convolutional Network) [19] was introduced to handle three-dimensional spatial features in data, effectively capturing spatiotemporal characteristics, but it requires significantly more computation and resources compared to 2D convolution. Therefore, we employ the TSM (Temporal Shift Module) [20] based on 2D CNNs in this study. TSM shifts a portion of the feature channels from previous and subsequent frames along the temporal dimension, facilitating temporal information exchange without adding additional computational burden.

**Figure 1:**
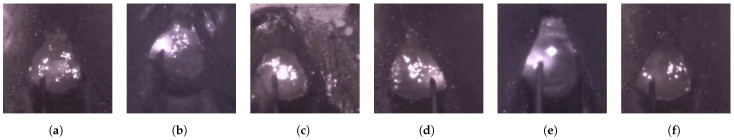
Images of the melt pool in different scenes. (**a**,**b**) are differences in the shape of the melt pool; (**c**,**d**) are differences in the camera viewpoint; and (**e**,**f**) are differences in size.

Due to variations in the camera’s focal length, different installation angles, and welding scenarios, the position and size of the molten pool within the images are not fixed. Additionally, the redundant background information in the molten pool images poses challenges for welding defect detection. To address this, we segment the molten pool region in the images during preprocessing and crop the images based on the segmentation results. However, as shown in Figure 1, the size and position of the molten pool in the cropped images remain inconsistent, which complicates defect recognition. Inspired by the spatial pyramid structures in [21,22,23] and the feature fusion modules in [24,25], we added a MFF (Multi-Scale Feature Fusion) module at the head of the network. This module increases the receptive field of the network, enabling it to capture multi-scale features of the molten pool region. Additionally, we found that the attention mechanism further aids in weighting the features, enhancing the propagation of molten pool region features.

**Figure 2:**
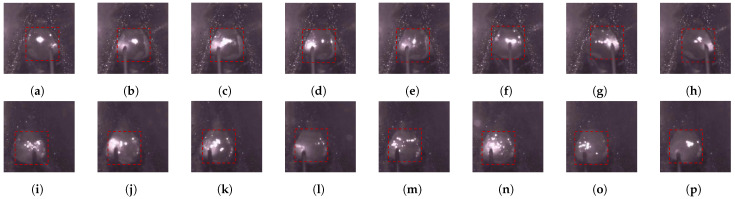
Eight time intervals of molten pool surface changes. (**a**–**h**) is the normal molten pool image of molten pool surface spot changes; (**i**–**p**) is the molten pool image of molten pool surface spot changes in the case of porosity defects.

In the Gas Metal Arc Welding (GMAW) process, porosity defects often arise due to insufficient shielding gas flow, high humidity in the air, unclean weld seams, or moisture contamination on the welding plates. When dense porosity occurs internally, the shape of the molten pool and the surface light spots exhibit erratic and unstable changes. In contrast, a normal molten pool and its surface light spots show stable variations. Figure 2 illustrates the differences in molten pool behavior between porosity defects and normal conditions. This study focuses on porosity defects, aiming to extract critical information from the spatial characteristics of surface light spots and the dynamic features of molten pool image sequences to enhance the identification of welding defects. Our main contributions are as follows:

(1) We propose a lightweight multi-scale feature fusion module that improves feature propagation and fusion, capturing features from different scales and directions in molten pool images. The module enhances the model’s expressive capability and its adaptability to molten pool size variations.
(2) We introduce an attention module that combines features from different directions and attention mechanisms to improve the model’s ability to recognize both large objects and fine details, facilitating better propagation of features in the molten pool region.
(3) We establish a mapping model that links the temporal dependencies in molten pool image sequences to welding defects, leveraging the dynamic characteristics of the molten pool during the welding process to achieve efficient defect recognition.

## 2. Methods

**Figure 3:**
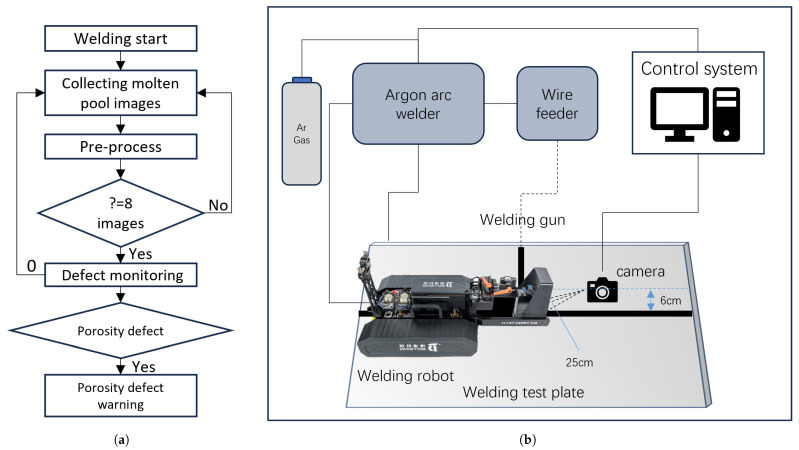
Welding defect recognition process and melt pool vision system schematics: (**a**) welding defects monitoring process; (**b**) schematic diagram of molten pool vision system.

Figure 3 illustrates the process of welding defect monitoring and identification. Images captured by the molten pool vision system are first preprocessed, and then a sequence of 8 consecutive frames is fed into the defect monitoring model. The model identifies the type of defect and issues a warning if necessary.

### 2.1. Molten Pool Visual System

This study collected data and conducted related experiments using the trackless crawling welding robot and the molten pool camera vision system developed by Beijing BOTSING Technology Co., Ltd., Beijing 100176, China. Figure 3 shows the molten pool vision system equipment used in this study, including the molten pool camera, the trackless crawling welding robot, and the industrial control computer.

The molten pool camera captures videos with a 640 × 512 pixels resoulution at a frame rate of up to 30 FPS. The camera is controlled by an industrial control computer, allowing for flexible recording. The experiments were conducted within a GMAW welding system. During GMAW welding, arc light can obscure many details in the weld pool image, decreaseing image quality. This study utilized the company’s second-generation molten pool camera, which can filter out most of the arc light, thereby revealing more details in the molten pool images and reducing the negative impact of arc light on image quality.

### 2.2. Network Architecture

In the field of welding, there are stringent requirements for weld quality. Additionally, detecting welding defects in real-time and making adjustments promptly can significantly improve weld quality. To address this, we propose a lightweight welding defect detection model that processes video sequences as input. These sequences are represented as $A\in R^{NTCHW}$, where N is the batch size, T is the temporal dimension, C is the number of channels, and H and W are the spatial dimensions. In our model, a CNN extracts enhanced and effective features from each frame of the molten pool images, and the TSM module is used to capture the temporal dependencies of these features, enabling accurate identification of welding defects.

**Figure 4:**
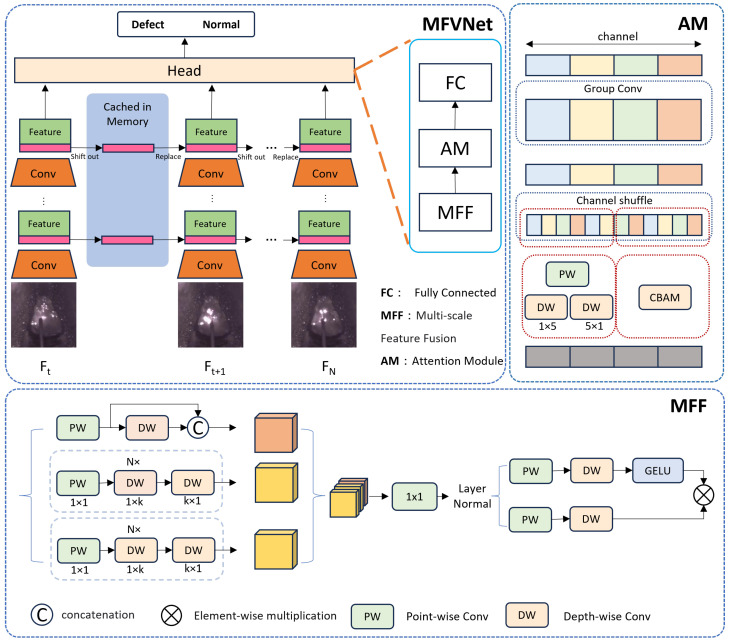
Structure of the proposed model MFVNet. the MFVNet consists of a backbone network and a header, with multi-scale feature fusion module and attention module embedded in the header and temporal shift module (temporal shift) in each layer.

**Figure 5:**
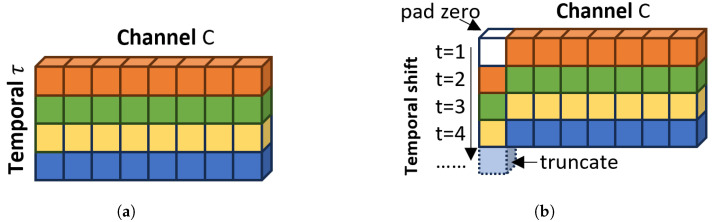
The Temporal Shift Module (TSM) performs efficient temporal modeling by shifting the feature map along the time dimension. The unidirectional TSM mixes the past frame with the current one. (**a**) The original tensor without shift. (**b**) Temporal shift (uni-direction).

As shown in Figure 4, the Multi-Scale Feature Fusion Network based on Molten Pool Video (MFVNet) consists of a backbone and a head. The backbone processes the original molten pool image sequence to extract feature information. The head then further processes these features and performs the final classification. The head comprises three main components: a multi-scale feature fusion (MFF) module, an attention module (AM), and a fully connected layer. The MFF module has three branches and extracts and fuse feature information from the feature maps. Each layer stacks convolutions with different kernel sizes to further process the extracted molten pool features, combining branches with different receptive fields. The MFF module allows the model to detect targets at various scales. Additionally, we incorporate an attention module to enhance the model’s ability to recognize both large objects and fine details. This is achieved by applying convolutional block attention module (CBAM) [26] attention and stacked row-column convolutions to the channel-shuffled features, weighting them for more effective feature processing. To further improve the accuracy of welding defect detection, we add Temporal Shift Modules (TSM) [20] after each layer of the backbone and after the MFF and AM modules. These TSM modules capture dynamic feature information from the video sequence, which is crucial for recognizing welding defects. Given the real-time requirements of welding defect detection, we use a unidirectional TSM module, as shown in Figure 5, to shift features from the previous frame to the current frame.

**Figure 6:**
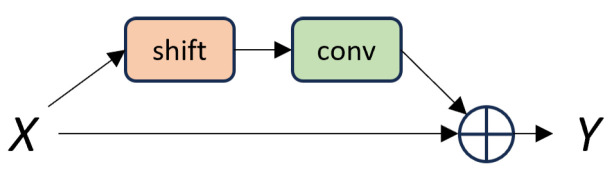
Residual TSM.

In the backbone section, we designed a relatively lightweight backbone network due to the high real-time requirements of industrial applications for welding defect detection. We chose the MobileNetV2 [9] as the backbone, which utilizes depthwise separable convolutions and inverted residual structures. This design maintains a lightweight architecture while achieving excellent performance, extracting features from molten pool images to enhance welding defect recognition. Similar to the approach in [20], we integrated TSM modules into each backbone layer, as illustrated in Figure 6. The TSM modules use residual shifts to fuse temporal information within the residual branches, further improving the model’s ability to detect welding defects.

In this study, we propose a lightweight Multi-Scale Feature Fusion (MFF) module, which integrates concepts from feature pyramids to enhance the model’s ability to handle multi-scale features. The MFF module consists of three branches and a feature fusion component, each branch using convolutions with different kernel sizes to improve the model’s ability to extract multi-scale features. The first branch comprises a pointwise convolution layer (PW) and a depthwise convolution layer (DW). After the pointwise convolution, the number of feature channels is reduced to half of the input. Then, the original number of channels is restored through a residual structure and group convolution at the feature layer. This operation significantly reduces the number of parameters and accelerates the model’s inference speed. Inspired by the feature pyramids in [21,22,23], the second and third branches also aim to capture features at different scales by increasing the receptive field. However, unlike those works, we do not use dilated convolutions to achieve varying receptive field sizes. Instead, we employ pointwise convolutions combined with row and column group convolutions of different kernel sizes, capturing features at different scales in various directions. In each branch, we stack these operations to enable the model to capture both detailed and global information at the same level, thereby enhancing the model’s ability to handle complex backgrounds and intricate details.

Before feature fusion, we set a relatively small batch size due to the equipment’s limitations. To mitigate this impact on model performance, we introduced a Layer Normalization (LN) layer. The LN layer normalizes all features within each sample, ensuring that features from different sources have similar distribution ranges, thereby eliminating the influence of batch size on the model’s performance. In the feature fusion module, we combined depthwise convolution layers with a 3 × 3 kernel size and pointwise convolution layers with a 1 × 1 kernel size. This setup allows for efficient information fusion and encoding. We also implemented a gating mechanism, adding an extra path after the GELU activation function as a gate. This mechanism facilitates the effective propagation and fusion of features, enabling the model to focus on finer details.

**Figure 7:**
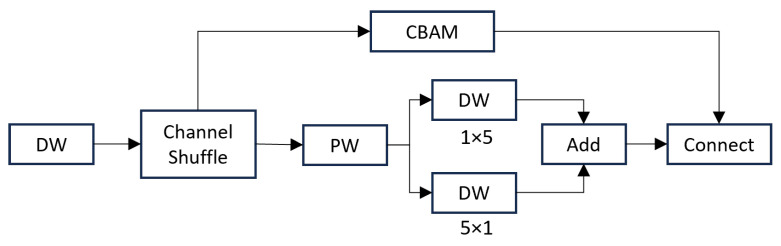
Attention mechanism flowchart.

Attention mechanisms are widely used in deep learning to recognize large objects and distinguishing between foreground and background. To enhance the model’s performance, we designed a custom attention module. As shown in the Figure 7, we divided the feature channels into different groups using grouped convolutions, followed by a channel shuffle operation to increase interaction between different groups in the grouped convolution. In one branch, we employed the Convolutional Block Attention Module (CBAM), while in another branch, we used pointwise convolution combined with row and column group convolutions. CBAM is a lightweight attention mechanism that combines channel attention [27] and spatial attention [28] and can be flexibly integrated into CNN networks [26]. CBAM assigns higher weights to important targets, allowing the model to focus more on them while suppressing irrelevant features like background noise by assigning them lower weights. This helps the model effectively filter out background interference and focus on the critical features of the molten pool. Combining mixed attention with features from different directions improves the model’s capability to detect fine details, enhancing its ability to detect subtle changes, such as size and shape.

### 2.3. Loss Function

Cross-entropy loss is a commonly used loss function in classification problems, particularly in deep learning models like Convolutional Neural Networks (CNNs). It measures the difference between the predicted probability distribution and the actual probability distribution. In binary classification problems, the model’s output is typically a probability value representing the likelihood that a sample belongs to the positive class. The cross-entropy loss can be expressed as

(1) $$Loss=\frac{1}{N}\sum_{i}^{N}-\left[y_{i}\log\left(p_{i}\right)+\left(1-y_{i}\right)\log\left(1-p_{i}\right)\right].$$

where $y_{i}$ denotes the truth value of the sample *i*, with 1 for the positive class and 0 for the negative class. $p_{i}$ denotes the probability that the sample *i* is predicted to be in the positive class.

## 3. Experiments Design

### 3.1. Dataset

This study used GMAW welding, two types of welding wire (solid wire and flux-cored wire), and 980 high-strength steel with welds having different gaps and groove angles, and a molten pool vision system was employed to collect regular and porosity defect molten pool videos from horizontal and vertical welding on 30 welding plates. The data collected from four plates in both the normal and porosity defect samples were used as the test set. The captured videos had a 640 × 512 resolution at 30 frames per second. The videos were segmented into 1 s samples, with one sample taken every 4 s. Eight frames were evenly selected from each sample, with approximately 600 normal molten pool samples and 600 porosity defect samples. Since the molten pool images include parts of the weld seam, base metal, welding torch, and molten pool, with the molten pool region being the primary focus for detecting welding defects, this region occupies only a small portion of the image. High resolution and unnecessary background details can negatively impact the performance and speed of the neural network. Therefore, the molten pool region was segmented in each of the eight frames per sample. Based on the segmentation results, the molten pool region was cropped from the original image with a 1:1 aspect ratio, and the size was then resized to 224 × 224 pixels without altering the original aspect ratio of the molten pool region. This resizing preserves the image details and minimizes the impact on accuracy while improving processing speed by four times. The resulting dataset was named WELDPOOL.

### 3.2. Test Environment

In the experiment, we used a desktop computer with a GeForce RTX 3060 GPU and a 12th Gen Intel(R) Core(TM) i9-12900H CPU running Windows 11 operating system. The WELDPOOL dataset was utilized, with the dataset divided into training, validation, and test sets in a ratio of 6:1:3. The training parameters were set as follows: an initial learning rate of 0.0001, a weight decay coefficient of 0.00005, and, since we used pre-trained weights from MobileNetV2, we followed the settings in [20] and set the number of training epochs to 50. Parameters were updated using the gradient descent method. The batch size for each training iteration was set to 4 due to GPU limitations.

### 3.3. Performance Metrics

To evaluate the effectiveness of the proposed model in identifying porosity defects objectively, this study assesses the model from both performance and real-time capability perspectives. We use accuracy (Top-1 and Top-5), recall, and F1-score as the key metrics for performance evaluation. We consider the number of parameters and computational complexity for real-time capability as evaluation criteria. Following the settings in [20], we also include latency and throughput as real-time evaluation metrics. Latency refers to the time taken to process a single sample during inference, which in this study is the delay in processing a sequence sample consisting of 8 frames. Latency directly reflects the model’s real-time capability. Conversely, throughput indicates the amount of data that can be processed per second, serving as an essential metric for evaluating the model’s efficiency.

### 3.4. Experiments

The experiments were conducted in the environment specified in Section 3.2. First, we conducted benchmark tests to validate the proposed approach and the effectiveness of the model. To further assess the effectiveness of the proposed modules, we performed ablation experiments using the TSM model with MobileNetV2 as the backbone. Specifically, the Multi-Scale Feature Fusion (MFF) module and the Attention Module (AM) were tested as validation components, and their impact was further illustrated using confusion matrices. Next, to verify that the dynamic features of the molten pool can enhance the model’s recognition capability and to assess the impact of sample frame count on the model, we compared the model’s result under different sample frame counts. Additionally, to explore the effect of different backbones on the model’s performance and real-time capability, we conducted comparative experiments by replacing the backbone with several lightweight alternatives and comparing them with the proposed model. Finally, to demonstrate the superiority of our algorithm, we compared it with several existing video classification algorithms. To further verify the generalizability of the proposed algorithm, we also conducted classification tests on the UCF101-24 and JHMDB subsets of the action recognition datasets UCF101 [29] and HMDB51 [30] to evaluate the performance of the proposed method.

## 4. Experiments Results

### 4.1. Benchmark Testing

This section primarily discusses the comparison between the proposed model and the baseline model. We enhance defect recognition by utilizing the dynamic features of the molten pool. To improve the model’s adaptability to different molten pool scales, we apply multi-scale feature fusion. Additionally, we use attention mechanisms to strengthen the model’s ability to identify the molten pool region. Based on this approach, we set up a baseline model that includes TSM, a spatial pyramid module, and CBAM.

As shown in Table 1, the proposed model has a lower parameter count and computational complexity compared to the baseline model. It achieves higher accuracy in Top-1 classification, although slightly lower in Top-5 accuracy. This indicates that the effective stacking of row and column group convolutions at different scales, combined with efficient feature fusion, is superior to using dilated convolutions with various dilation factors and pooling layers. This approach allows the proposed model to extract multi-scale features from different directions accurately and effectively while maintaining lower parameters and computational load. Moreover, incorporating the attention mechanism within the channel shuffle branch proves to be more effective than using the CBAM attention mechanism alone. Additionally, the proposed model demonstrates a significant advantage in processing speed.

Furthermore, we evaluated the proposed model using seven-fold cross-validation. The model achieved an average accuracy of 97.16%, with a standard deviation of 0.24%. This result indicates high accuracy and remarkable stability across different folds, demonstrating the model’s generalization ability.

### 4.2. Ablation Study

The results of validating the Multi-Scale Feature Fusion module (MFF) and the Attention Module (AM) on the TSM baseline model with MobileNetV2 as the backbone are shown in Table 2. The experimental results indicate that when the proposed Attention Module (AM) is integrated directly into the baseline model, the Top-1 accuracy improves by 0.258%, and the recall rate increases by 0.2%. The results also demonstrate that the AM module helps the model leverage more effective features, significantly enhancing overall performance. The model incorporating the Multi-Scale Feature Fusion module (MFF) outperforms the baseline model on our dataset due to its enhanced capability for multi-scale feature extraction. The Top-1 accuracy improved by 1.292%, and the recall rate and F1 score of the baseline model and the model with only the AM module were also lower than those of the TSM model with the MFF module. Consequently, we integrated both modules into the TSM model to improve performance. Compared to other model combinations, MFVNet demonstrated superior results across the board. Therefore, from these experiments, we can conclude that the proposed Attention Module (AM) and Multi-Scale Feature Fusion module (MFF) effectively capture molten pool features and are well-suited for real-time monitoring and identification of welding defects in our welding scenarios.

**Figure 8:**
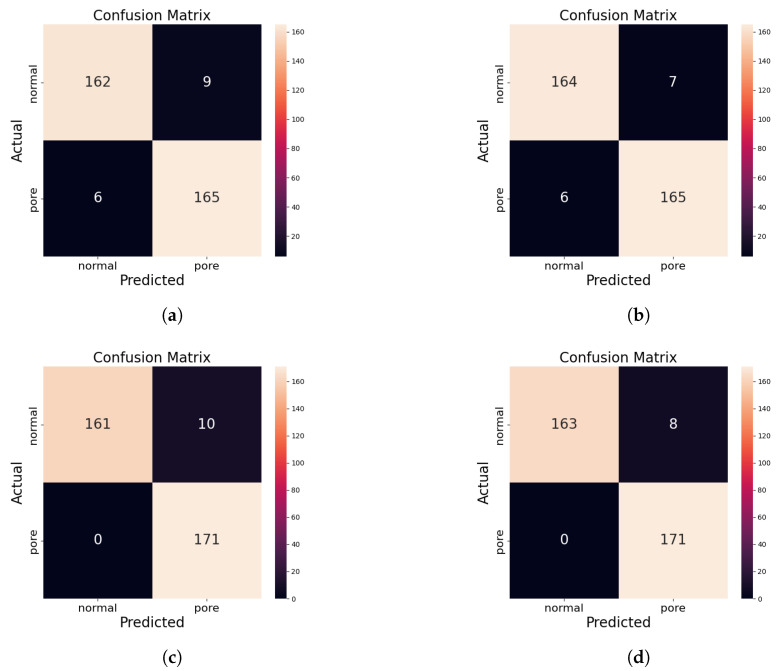
Confusion matrix results for different configuration models on the WELDPOOL test set. (**a**) TSM. (**b**) TSM-AM. (**c**) TSM-MFF. (**d**) MFVNet.

To further demonstrate the effectiveness and impact of each module, we also calculated the confusion matrix for the inference results under each model configuration, as shown in Figure 8. In the confusion matrix, each column represents the predicted class, with the total number in each column indicating the count of data predicted as that class. Each row represents the actual class, with the total number in each row showing the number of instances of that class. The experimental results indicate that, regardless of the model configuration, a minimal number of normal samples are misclassified as porosity defects, possibly due to other unstable factors in the welding process causing instability in the molten pool surface light spots. However, after integrating the proposed modules, the model’s misclassification rate decreases, demonstrating that the MFF and AM modules enhance the model’s ability to identify welding defects.

To further investigate the causes of model misclassification, we conducted tests on normal molten pool samples with different conditions: the presence or absence of spatter, variations in shape, and differences in size. Case1 represents no spatter, while Case2 represents the presence of spatter. The results are shown in the Table 3. It can be observed that variations in shape and size have minimal impact on the model’s performance, while the presence of spatter has a more significant effect. This is likely due to the bright spots caused by metal spatter, which interfere with the model’s ability to accurately recognize the true state of the molten pool. Therefore, improving the model’s robustness to spatter interference remains a critical direction for further optimization.

**Figure 9:**
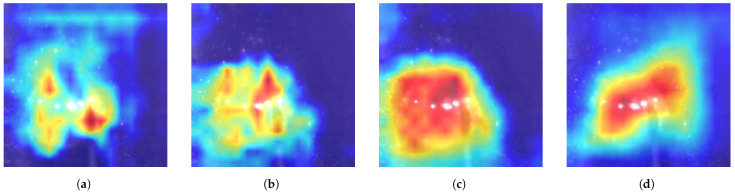
In Class Activation Map (CAM), the importance of features is visualized using different colors, where the heatmap colors range from blue, yellow, orange to red, representing the lowest to highest feature importance, respectively. Class Activation Map (CAM) results for different configuration models on the WELDPOOL test set: (**a**) TSM; (**b**) TSM-AM; (**c**) TSM-MFF; (**d**) MFVNet.

Figure 9 presents the class activation map (CAM) visualizations for each model configuration. As shown in Figure 9b,c, the proposed MFF and AM modules significantly enhance the model’s ability to identify the molten pool spots. Furthermore, Figure 9d demonstrates that combining the MFF and AM modules enables the model to focus more effectively on critical regions, thereby improving detection accuracy.

### 4.3. Impact of Sample Frames Number on the Model

This section discusses the impact of different sample frame counts on the model’s real-time performance and accuracy. When the sample frame count is 1, only a single frame is in the time series, and no feature shifting is applied. Unidirectional feature shifting is used for sample frame counts ranging from 2 to 16. To ensure a fair comparison, we use the inference time of a single frame as a metric for real-time performance in this experiment.

**Figure 10:**
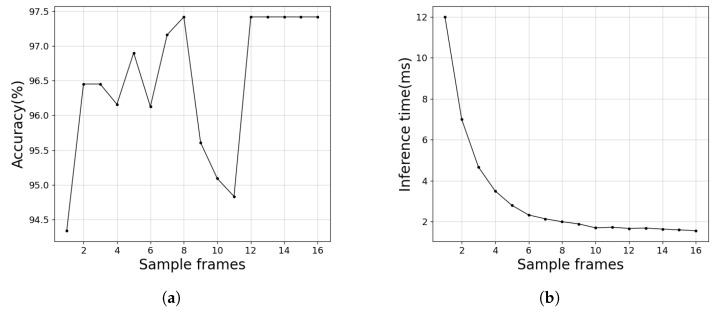
Impact of different sample frame sizes on the real-time and performance of the model. (**a**) Accuracy of models with different sample frame sizes. (**b**) Time to reason about single-frame images for different sample frame number models.

As shown in Figure 10a, when the sample frame count is between 2 and 16, the model’s performance improves compared to when the frame count is 1, indicating that dynamic features in the molten pool images enhance the model’s ability to identify welding defects. Furthermore, when the sample frame count reaches 12 to 16 frames, the model’s ability to extract dynamic features from the molten pool video stabilizes. From a real-time perspective, as illustrated in Figure 10b, the inference time per image decreases as the sample frame count increases. However, after the sample frame count reaches 8, the improvement in inference speed becomes negligible. Therefore, the experimental results suggest that with a sample frame count of 8, the model’s accuracy and real-time performance meet the requirements for real-time welding defect monitoring in industrial applications.

### 4.4. Comparison with Other Backbone

The comparison results with two other popular lightweight backbone networks, ShuffleNetV2 [31] and GhostNetV2 [32], are shown in Table 4. The model using MobileNetV2 as the backbone performs the best across several evaluation metrics, including Top-1 accuracy, recall, F1 score, and inference latency. While GhostNetV2 and MobileNetV2 achieve the same accuracy, GhostNetV2 has a significantly slower inference latency of 27ms compared to MobileNetV2’s 16ms, likely due to the higher number of parameters in GhostNetV2. Although ShuffleNetV2 matches MobileNetV2 in inference latency, it falls short in Top-1 accuracy. We speculate that the extensive channel shuffle operations in ShuffleNetV2, while enhancing information flow, may have slightly compromised its feature extraction capability on our dataset. The model with MobileNetV2 as the backbone achieves an accuracy of 97.416% with an inference latency of 16ms, meeting the accuracy and real-time requirements necessary for monitoring welding defects during the welding process.

### 4.5. Comparison with Other Methods and Dataset

In this section, we analyzed the work presented in this paper in comparison to the latest research on molten pool analysis. For a fair comparison, we made adjustments to AMSegNet (additive manufacturing–SegNet) [33]. Specifically, we added Temporal Shift Modules to the convolutional layers after each downsampling and upsampling step to better handle time-series data. Additionally, we modified the final output to categorical. As shown in the Table 5, although AMSegNet demonstrates excellent performance in terms of top-1 accuracy, recall, and F1 score, its computational complexity is significantly higher, reaching 64.003 G FLOPs, with 17.064 M parameters. This results in a higher latency of 71 ms and a lower throughput of only 14.1 V/s. Although AMSegNet uses lightweight CNN modules, modifying its input to image sequences considerably increased the number of parameters and computational load. However, with profit from the attention mechanism, AMSegNet still achieved outstanding performance in detecting molten pool defects.

The experimental results of CNN+LSTM, C3D [19], Video Swin (Tiny) [34], and our proposed MFVNet across different datasets are shown in Table 6. The performance trends of the models are consistent across all three datasets: they perform well on our custom WELDPOOL dataset and the UCF101-24 dataset but show weaker performance on the JHMDB dataset, possibly due to the weaker temporal relationships in JHMDB. Our model has been optimized for the WELDPOOL dataset, primarily by capturing features from molten pool images at different scales and improving defect recognition through temporal modeling. This optimization also leads to strong performance on the action recognition dataset UCF101-24, with similar improvements observed in the CNN+LSTM model when these modules are incorporated. On our custom dataset, the proposed modules enable our model to achieve the best performance, with significantly lower inference latency compared to other models. The Video Swin model’s performance is moderate, possibly be due to the reduced number of layers in the tiny version. Although our model demonstrates a clear advantage in inference latency on the UCF101-24 dataset, its accuracy is only slightly better than that of the CNN+LSTM model. As noted in the literature [20], methods that integrate temporal fusion across all layers generally outperform those like CNN+LSTM, which only apply temporal fusion in later feature extraction stages. On the JHMDB dataset, our model’s accuracy is 0.989% lower than that of Video Swin, but it still outperforms the other two models and achieves the best inference latency.

In summary, our proposed MFVNet demonstrates excellent performance across multiple datasets, with a significant advantage in inference speed. While its accuracy on certain datasets, such as UCF101-24, is slightly lower than the top-performing model (e.g., Video Swin), MFVNet’s substantial advantage in inference latency, combined with superior accuracy on our custom welding defect dataset, makes real-time welding defect monitoring feasible during the welding process.

## 5. Discussion

This study presents MFVNet, a video-based model for welding defect classification. The model uses the lightweight MobileNetV2 as the backbone for feature extraction and incorporates lightweight temporal shift modules in each layer, making the model more efficient. To address the characteristics of our molten pool images, we added a Multi-Scale Feature Fusion (MFF) module, which captures features from different scales and directions of the molten pool and integrates them across feature channels. The structure allows the model to better capture temporal dependencies within the sequence. Additionally, we introduced an attention mechanism module (AM) that combines attention with features from different directions, enabling the model to focus on the most important features. The dataset used in this study was created using a trackless crawling welding robot from Beijing BOTSING Technology, with molten pool videos captured by a molten pool camera, consisting of 1200 samples, with eight frames uniformly sampled from each video for training and validation. Experimental results demonstrate that the proposed model achieves low latency (16ms per sample) and high throughput (62.5 video samples per second), meeting the real-time requirements of practical applications. The model also achieved a welding defect recognition accuracy of 97.416%, laying a solid foundation for molten pool video-based welding defect detection. Furthermore, experiments on the UCF101-24 and JHMDB datasets indicate that our model is generalizable.

While our model has shown high performance and good real-time capabilities on our custom dataset, some limitations remain. The current algorithm focuses on identifying potential porosity defects, but further exploration is needed to extend it to identify a wider range of welding defects. Although we built a dataset with 1,200 samples, deep learning models typically benefit from larger datasets for improved generalization and robustness. Future research should delve deeper into the model, welding defects, and molten pool characteristics, and collect more diverse and larger-scale welding defect video data to enhance the model’s performance.

## Figures and Tables

**Table 1:** Results for different benchmark configurations on the WELDPOOL dataset.

Moudle	#Test-Top1	#Test-Top5	#Param	#FLops	#Thrput
Baseline	96.833%	**99.911%**	13.108 M	6.912 G	22 ms
Baseline-AM	96.997%	98.95%	13.108 M	6.914 G	22 ms
Baseline-MFF	97.322%	98.962%	4.839 M	**3.669 G**	**15 ms**
MFVNet	**97.416%**	99.728%	4.840 M	3.671 G	16 ms

**Table 2:** Results of different model configurations on the WELDPOOL dataset.

Moudle	#Test-Top1	#Test-Top5	#Recall	#F1	#Δacc
TSM	95.866%	98.325%	95.9%	95.9%	0%
TSM-AM	96.124%	98.85%	96.1%	96.1%	0.258%
TSM-MFF	97.158%	98.9%	97.2%	97.2%	1.292%
MFVNet	**97.416%**	**99.728%**	**97.4%**	**97.4%**	**1.55%**

**Table 3:** Results of the proposed model on normal molten pool samples with different conditions.

Conditions	#Case1	#Case2
splashes	99.617%	94.839%
shapes	99.774%	99.735%
size	99.769%	99.803%

**Table 4:** Results of the proposed model using different backbone on the WELDPOOL dataset.

Backbone	#Test-Top1	#Test-Top5	#FLOPs	#Param	#Latency	#Thrput
MFVNet (ShuffleNetV2)	96.641%	99.128%	**2.435 G**	**4.260 M**	**16 ms**	**62.5 V/s**
MFVNet (GhostNetV2)	**97.416%**	**99.728%**	3.619 G	9.048 M	27 ms	37 V/s
MFVNet (MobileNetV2)	**97.416%**	**99.728%**	3.671 G	4.840 M	**16 ms**	**62.5 V/s**

**Table 5:** The latest research on the WELDPOOL dataset.

Model	#Top1	#recall	#F1	#FLOPs	#Param	#Latency	#Thrput
AMSegNet	97.158%	97.2%	97.2%	64.003 G	17.064 M	71 ms	14.1 V/s
MFVNet	**97.416%**	**97.4%**	**97.4%**	**3.671 G**	**4.840 M**	**16 ms**	**62.5 V/s**

**Table 6:** Experimental results of different models on different datasets.

Model	WELDPOOL	UCF101-24	JHMDB
CNN-LSTM	96.425%	96.596%	69.681%
	(50.9 ms)	(52.6 ms)	(52.2 ms)
C3D	95.85%	98.234%	68.989%
	(123.89 ms)	(119.53 ms)	(126.48 ms)
video swin	96.37%	**98.338%**	**71.372%**
	(62.26 ms)	(55.49 ms)	(66.14 ms)
MFVNet(our)	**97.416%**	97.819%	70.383%
	**(16 ms)**	**(13 ms)**	**(17.5 ms)**

## Data Availability

The data presented in this study are available on request from the corresponding author.
